# Dermatomyositis Masquerading As Generalized Body Swelling: A Case Report

**DOI:** 10.7759/cureus.38895

**Published:** 2023-05-11

**Authors:** Neethu Sunny, Kritartha Kashyap, Arjun Kumar, Ashwin Parchani, Minakshi Dhar

**Affiliations:** 1 Geriatrics, All India Institute of Medical Sciences, Rishikesh, IND; 2 Internal Medicine, All India Institute of Medical Sciences, Rishikesh, IND

**Keywords:** anti-tif1γ antibodies, inflammatory myopathy, periorbital edema, dermatomyositis, generalized edema

## Abstract

Dermatomyositis (DM) is a systemic autoimmune disease that primarily affects the skin and muscles. The hallmark skin manifestation is a violaceous rash on the face, neck, shoulders, upper chest, and extensor surfaces of the arms and legs, which is often accompanied by edema and can be exacerbated by exposure to sunlight. Generalized limb edema and dysphagia are rare presentations of dermatomyositis. Here we present a case of a 69-year-old woman presenting with generalized limb swelling, periorbital swelling, and dysphagia which was diagnosed as dermatomyositis based on a combination of clinical, laboratory, and imaging findings. The patient had an absence of complaints of limb weakness and a predominance of complaints of edema and dysphagia which posed a diagnostic challenge. The patient was treated with high-dose steroids and immunosuppressive therapy, leading to a significant improvement in her symptoms. Edematous dermatomyositis has been associated with underlying malignancy in 25% of the cases and this warrants close follow-up and malignancy screening for such patients. In some cases, subcutaneous edema might be the only manifestation of the disease. This case underscores the importance of recognizing DM as a potential differential diagnosis in patients presenting with generalized edema and dysphagia, particularly in the initial absence of classic skin findings. This rare presentation of dermatomyositis may be a hallmark of a severe form of the disease and requires prompt recognition and aggressive treatment.

## Introduction

Dermatomyositis (DM) is a systemic autoimmune disease that primarily affects the skin and muscles. DM has a predilection to females in their fifth to seventh decade of life [1]. It is characterized by a chronic inflammatory response that involves the microvasculature of the skin and muscle tissue [1]. The pathogenesis of DM is believed to involve both genetic and environmental factors, with several potential triggers including infections, medications, and ultraviolet radiation or underlying malignancy [2]. The hallmark skin manifestation is a violaceous rash on the face, neck, shoulders, upper chest, and extensor surfaces of the arms and legs, which is often accompanied by edema and can be exacerbated by exposure to sunlight [3]. The muscle involvement in DM presents as a symmetric proximal muscle weakness, typically affecting the neck, shoulders, hips, and thighs. Other systemic features of DM may include dysphagia, interstitial lung disease, and cardiac involvement [4]. Amid such heterogeneous presentation, it is quite uncommon for a patient of dermatomyositis to present with generalized edema. Here the authors report a 69-year-old lady who presented with generalized edema and dysphagia, who after thorough evaluation was diagnosed to be a case of edematous dermatomyositis. It is an atypical presentation of the disease with less than 30 adult cases reported so far [5]. This form of dermatomyositis tends to be more aggressive and is associated with a higher risk of malignancy [6].

## Case presentation

A 69-year-old woman presented with complaints of generalized body swelling for four months, which started as periorbital swelling around the right eyelid and eventually spread to the left eyelid over the course of three days. The swelling gradually progressed over both lower limbs over the course of the following two months, then her upper limbs, which limited her mobility and forced her to become bed-bound and dependent. She first experienced trouble swallowing solid boluses of food two months into the illness. Over the course of a month, the dysphagia worsened and she started experiencing a "choking" sensation while attempting to eat semi-solid food. She also noticed skin changes in the form of darkening over the dorsum of her nose and ears, followed by periorbital regions, neck, and upper chest.

**Figure 1:**
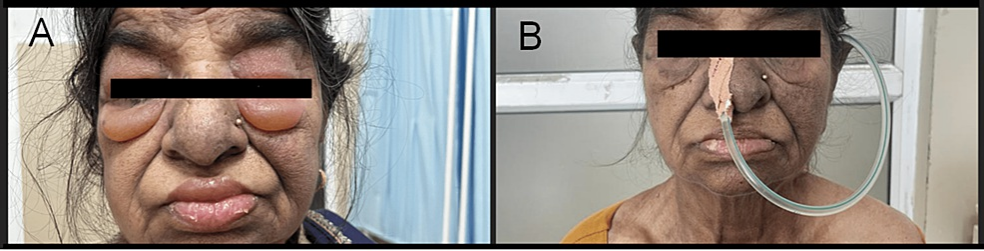
Before and after treatment photographs of the patient with dermatomyositis and generalized edema. Photograph of the patient (A) before treatment showing marked periorbital edema and (B) after treatment with high-dose steroids and immunosuppressive therapy, demonstrating a significant reduction in periorbital edema and lip swelling. The heliotropic rash is better appreciated in 1B after a reduction in periorbital edema following therapy.

**Figure 2:**
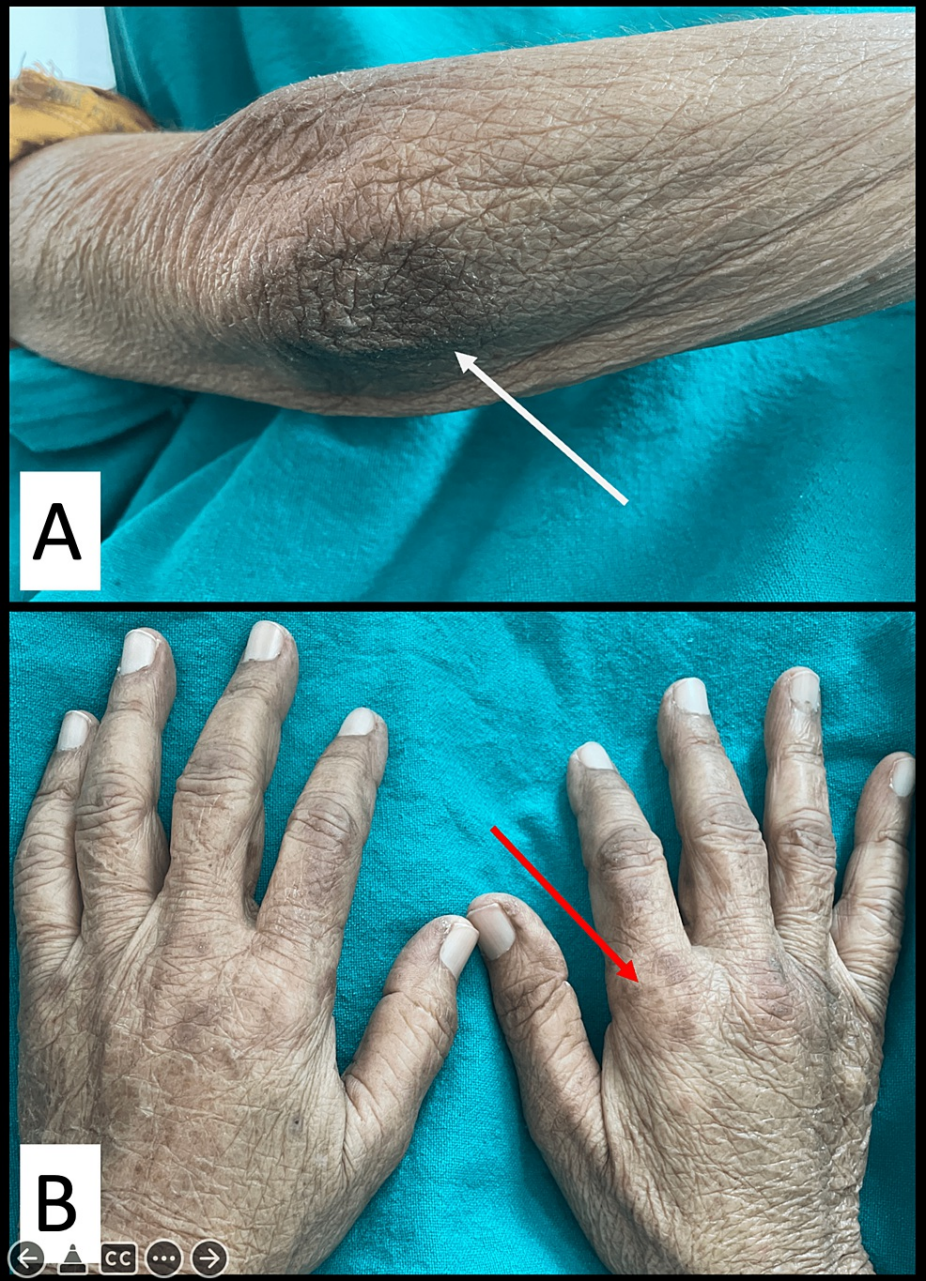
The appearance of Gottron's papules in the dermatomyositis patient. The images show (A) Gottron's papules on the patient's elbow and (B) Gottron's papules on the patient's knuckles. The papules became well-defined after the initiation of therapy and reduction in edema.

On examination, her vitals were stable. Bilateral periorbital edema with swelling around the lips, with bilateral painful non-pitting edema involving the bilateral lower limbs up to shin and bilateral upper limbs up to the forearms was noted (Figures 1A, 1B). Periorbital heliotropic rash and Gottron’s signs were distinctly appreciated (Figures 1A, 1B, 2A, 2B). Motor power was assessed to be Medical Research Council (MRC) grade 3/5 in bilateral upper limb proximal muscles and 2/5 in lower limb proximal muscles and neck extensors. The thigh and forearm muscles were tender on palpation.

**Figure 3:**
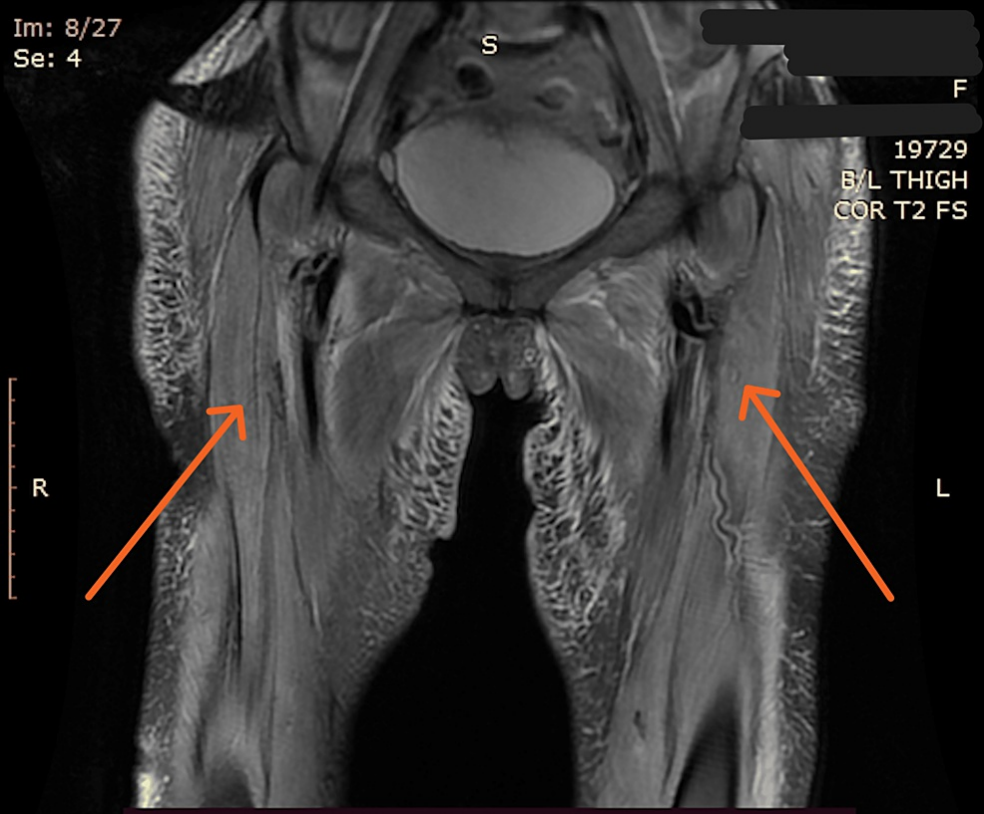
Coronal T2-weighted image of the pelvis and upper thigh shows bilateral symmetrical involvement of thigh muscles (arrows). Bilateral subcutaneous edema is visible.

With an initial impression of generalized edema, the patient was worked up to rule out relevant causes of generalized edema. Urine dipstick revealed no proteins, renal and liver function tests were within normal limits and a 2D echocardiography revealed an ejection fraction of 60% without any regional wall motion abnormality. Serum albumin was 3.1 g/dL and clinical evaluation didn’t divulge any evidence of malnutrition or protein-losing enteropathy. Thyroid profile and glycated hemoglobin were within normal limits. Serum aspartate transaminase (AST) was slightly elevated at 156 U/L (normal range: 20-40 U/L). A suspicion of myositis was kept in view of muscle tenderness and raised AST levels along with rash and dysphagia which were suggestive of an inflammatory/autoimmune etiology. Her creatine kinase (CK) was raised to 381 U/L (normal: 25-200 U/L) and her lactate dehydrogenase (LDH) level was 866 U/L (normal 105-333 U/L). Upper gastrointestinal tract (UGI) endoscopy ruled out any obstructive cause of dysphagia and contrast computed tomography of the abdomen and thorax revealed no signs of malignancy. Electromyography (EMG) recorded reduced interference pattern in the bilateral triceps, bilateral rectus femoris, and biceps long head. MRI thigh and bilateral arms revealed features of myositis in bilateral thigh and shoulder muscles with overlying diffuse subcutaneous edema (Figure 3). Her antinuclear antibodies were positive (1:100 titer), with extractable nuclear antibody (ENA) profile strongly positive for Ro-52 (3+). Autoimmune profile showed positivity for anti-transcriptional intermediary factor 1 (TIF-1γ) (2+). Based on the clinical findings and corroborative laboratory evidence, a final diagnosis of DM was made.

She was initiated on injection methylprednisolone pulse therapy (15 mg/kg) for three days followed by oral prednisolone (1 mg/kg) and subcutaneous methotrexate (15 mg once a week). Following steroid pulse, the patient showed some symptomatic improvement in her muscle power, her edema relatively reduced and her dysphagia slightly improved (Figures 1A, 1B). She was commenced on nasogastric feeding along with rehabilitative therapy for swallowing and was discharged on day eight of admission. She was followed up after two weeks by which her dysphagia had considerably resolved and nasogastric tube was hence removed. The patient became ambulatory and her prednisolone was gradually tapered to a target of 5 mg over three months. Cancer screening was performed with tumor markers, ENT evaluation for nasopharyngeal carcinoma, and pelvic examination were negative for any malignancy. The patient is currently on close follow-up with regular malignancy screening and immunosuppressive dose titration.

## Discussion

Dermatomyositis is one of the inflammatory myopathies caused by complement (C5b-9)-mediated microangiopathy that affects the skin and muscles [7]. Dysphagia in dermatomyositis patients is usually gradually progressive but severe dysphagia as an initial presentation of DM has been rarely reported [8]. Similarly, periorbital edema is common in dermatomyositis, especially in juvenile dermatomyositis [9], however, generalized edema caused by juvenile or adult dermatomyositis is uncommon and reported only in a few isolated cases [6]. The challenge in our case study was the absence of typical features of weakness in the history and the presence of edema and dysphagia as the chief presenting complaints. Subtle muscle tenderness on her proximal muscle groups and subsequent weakness in muscle power on clinical examination guided us toward the suspicion of dermatomyositis. The presentation was further complicated by the presence of a relatively low creatine phosphokinase-N acetyl-cysteine (CPK-NAC) level (two times elevated in our case) than is usually observed in the dermatomyositis (up to 50 times the upper normal limit). Dermatomyositis presenting as dysphagia has been reported to have a higher incidence of underlying malignancy as compared to DM without dysphagia [10]. We also did a contrast CT scan of chest and abdomen along with an upper GI endoscopy after consulting with gastroenterology to rule out any malignancy or acute structural obstruction or neurological cause. With an elevated CPK-NAC level, positive antinuclear antibody (ANA) and RO-52 and TIF-1γ, correlating with radiological and histopathological evidence, we reached the diagnosis of dermatomyositis presenting as anasarca and dysphagia.

Glucocorticoids remain the mainstay of the initial available treatment. Treatment with a short course of intravenous methylprednisolone (1 g daily for three days) is recommended prior to starting oral glucocorticoids in patients with severe weakness or comorbidities. In our case also, the patient was administered pulse methylprednisolone followed by oral steroids. Patients with severe weakness or other organ systems involvement, such as myocarditis or interstitial lung disease, as well as those who are at a higher risk of experiencing steroid-related complications like individuals with diabetes, osteoporosis, or postmenopausal women, or patients with myositis that is difficult to treat, require the addition of a second line immunosuppressive treatment for disease management [11]. In our case, the patient had exhibited a positive response to steroids and had experienced some early improvement in swallowing and muscle strength within a week of starting the treatment.

Persistent complaints of dysphagia in such patients of dermatomyositis might be considered for IV immunoglobulins infusion or other second-line immunosuppressants [12]. A recent research study utilizing both videofluoroscopic swallow study (VFSS) and manometry concluded that dysphagia observed in patients with inflammatory myopathy is primarily caused by weakened pharyngeal muscle contractions, which is linked to the weakness of the suprahyoid muscles [13]. Therefore, swallowing rehabilitation therapies like oropharyngeal exercises and compensation techniques would be more effective in managing the condition [14]. In another study, a significant association between the involvement of dysphagia and the existence of internal malignancy or anti-TIF-1γ Ab in dermatomyositis patients was found, where 10 out of 13 patients were positive for both anti-TIF-1γ and internal malignancy [15]. The presence of anti-TIF-1γ antibodies is considered to be a reliable predictor for the development of dysphagia in adult patients with dermatomyositis [16]. Our patient came out to be strongly positive for anti-TIF-1γ Ab although no evidence of malignancy was found.

Generalized edema is usually a rare finding in dermatomyositis cases. Increased capillary permeability secondary to complement activation and immune-complex deposition, leading to microvascular ischemia and subsequent disruption of the vascular endothelium leading to the development of edema has been proposed as a theory in some juvenile dermatomyositis cases [17].

## Conclusions

We report a rare case of Anasarca associated with dysphagia due to underlying dermatomyositis. Although dysphagia is a known complication of dermatomyositis, without the notable aggravation of other symptoms, it can make the diagnosis and treatment challenging. Similarly, inflammatory myopathies should be considered as a possibility, in cases of edema with unknown etiology. The literature suggests that individuals with dermatomyositis who experience anasarca either at presentation or during the course of their disease tend to have a particularly challenging course and may need aggressive immunosuppressive therapy. Identifying and treating these patients promptly is thus crucial. This highlights the significance of conducting a thorough medical history and relevant physical examination for all patients who present with such symptoms in order to consider any underlying conditions related to the patient's main concern and arrive at a diagnosis.
